# Laser-induced incandescence of iron nanoparticles: effects of laser-induced sintering and coalescence

**DOI:** 10.1007/s00340-025-08504-0

**Published:** 2025-06-11

**Authors:** Stephen Robinson-Enebeli, Christof Schulz, Kyle J. Daun

**Affiliations:** 1 Department of Mechanical and Mechatronics Engineering, University of Waterloo, 200 University Ave W, Waterloo, ON Canada; 2 EMPI, Institute for Energy and Materials Processes – Reactive Fluids, and CENIDE, Center for Nanointegration Duisburg Essen, University of Duisburg-Essen, 47048 Duisburg, Germany

## Abstract

While time-resolved laser-induced incandescence is a promising technique for characterizing metal nanoparticles in the gas phase, there remain several commonly observed and unexplained features in the data, including larger-than-predicted absorption cross-sections (excessive absorption) and faster-than-predicted cooling rates immediately following peak emission (apparent anomalous cooling). In the case of low melting point metals such as iron, laser-heated aggregates coalesce into spheres (i.e., fully sinter) before the peak of the LII signal is reached. Coalescence may affect the observed TiRe-LII signals in two ways: (i) the transition from aggregates to spheres reduces the absorption cross-section, which affects both the total absorbed laser energy and the intensity of the emitted incandescence in a wavelength-dependent manner; and (ii) surface energy is converted into sensible energy of the nanoparticles, which increases their peak temperature. While the revised LII model does not completely account for the rapid signal decay immediately following the peak signal, the predicted curves align more closely with the measured intensities from coalesced particles during the later cooling times.

**Supplementary Information:**

The online version contains supplementary material available at 10.1007/s00340-025-08504-0.

## Introduction

The growing demand for metal nanoparticles can be satisfied using scalable synthesis routes like spark discharge generation [1], laser ablation [2], photolysis [3], or plasma synthesis [4], which are in the gas phase. The functionality of metal nanoparticles in their end applications depends strongly on their size and morphology, presenting a need for online or in situ diagnostics to measure these attributes during particle synthesis.

Time-resolved laser-induced incandescence (TiRe-LII), an optical diagnostic mainly used to measure soot in combustion applications, may fulfill this need [5, 6]. In this technique, gas-borne nanoparticles in a probe volume are heated with a pulsed laser (often a Nd:YAG laser at 1064 nm) to incandescent temperatures. The nanoparticles emit thermal radiation in a size-dependent manner as they are heated and then return to the ambient gas temperature, primarily through evaporative and conductive cooling. Emissions are measured at two or more wavelengths using photomultipliers (PMT) equipped with suitable bandpass filters or streak cameras [5, 7].

Observational models derived from standard spectroscopic and heat transfer sub-models [8] are regressed to the temporally- and spectrally-resolved incandescence data or effective temperatures to infer the nanoparticle size distribution [9, 10] and volume fraction [11], as well as other properties that include the nanoparticle composition [12] and thermal accommodation coefficient (TAC) [9, 10]. While these experiments are well-established for soot-laden aerosols, several aspects of TiRe-LII measurements on metal nanoparticles are not fully understood. Notably, metal nanoparticles appear to absorb more laser energy than one would expect based on comparing the peak pyrometric temperature, laser fluence, and expected absorption cross-sections of the particle (“excessive absorption” [13]) and the particles seem to cool faster immediately after the laser pulse than can be explained through conventional heat transfer models (“anomalous cooling” [14]). These unexplained features indicate that the present understanding of LII for metal particles and thus the model used for data analysis is incomplete.

This point is exemplified by early LII studies on iron nanoparticles that erroneously applied the Rayleigh approximation to model the absorption cross-section (as is standard for soot) and furthermore assumed the refractive index to be wavelength-independent over the detection spectrum (~ 400–800 nm) [3, 15], which is not true for molten iron [16, 24]. Using this approach, Sipkens et al*.* [17] inferred TACs that were not consistent with expected values, which they attributed to neglecting the spectral dependence of the refractive index. Their later work [9] appeared to support the validity of wavelength-independent refractive index by reproducing the expected plateau temperature*,* the point at which the peak temperature becomes insensitive to increasing laser fluence due to increasing evaporative cooling. However, the peak pyrometric temperatures greatly exceeded what was predicted based on the absorbed laser energy, which they, along with others, postulated could indicate some other means of energy absorption, *e.g.*, an inverse Bremsstrahlung effect resulting from a laser-induced plasma that could envelop the nanoparticle [9, 18].

Talebi-Moghaddam et al*.* [13] investigated the limits of the Rayleigh assumption for metal nanoparticles and concluded that for iron nanospheres larger than ~ 30 nm in diameter, Mie theory needs to be applied to accurately predict the wavelength-dependent absorption cross-section of the nanoparticle. With this correction, the magnitude of the previously described excessive absorption effect was reduced substantially but not eliminated.

The deviation of the simulated TiRe-LII signals from measurements has also been attributed to interference from non-incandescence emission, including recombinative chemiluminescence [19] and plasma emission [20]. However, simulations show that plasma emission becomes important only at fluences above 9 mJ/mm^2^ at an excitation laser wavelength of 1064 nm, and detection wavelengths below 400 nm [20]. Recombinative chemiluminescence has been observed by Vander Wal et al*.* [19] at fluences greater than 12 mJ/mm^2^ at an irradiation wavelength of 1064 nm. Recombinative chemiluminescence is generally short-lived (< 100 ns [19]) compared to the LII signal and, hence, should only affect the peak LII signal.

While almost all previous LII studies on metal nanoparticles envision the aerosol as consisting of isolated nanospheres, in reality, solid metal nanoparticles are often aggregated. Our previous study showed that the aggregate structure can strongly enhance the absorption cross-section and cause significant variations in temperature within the aggregates at a given instant [14, 21]. In addition, aggregated nanoparticles can partially sinter (through the overlapping of primary particles) or fully coalesce into spheres as a result of laser heating, which will change their absorption cross-section [21]. We also hypothesized that the apparent anomalous cooling effect may be explained by sintering after the particle reaches its peak temperature, via the increased electrical and thermal contact between the primary particles [14] or by nonuniform aggregate heating that causes some particles to cool through evaporation which increases the expected cooling rate [21].

In this study, we assess these hypotheses using data from LII measurements on an aerosol of iron nanoaggregates formed through a spark discharge process. Measurements are interpreted using absorption cross-sections modeled using the multi-sphere T-matrix (MSTM) method (point contact), the discrete-dipole approximation (partially sintered), and Mie theory (fully sintered, *i.e.*, coalesced).

The remainder of the paper is organized as follows: Sect. 2 presents the experimental setup used in this work and Sect. 3 provides a summary of the TiRe-LII model and the modeling approach used to simulate the structure of aggregates and derive their absorption properties. In Sect. 4, we attempt to reproduce the measured pyrometric temperatures while incorporating sintering and coalescence into the LII model. Finally, we evaluate the robustness of prompt LII signals under conditions where apparent anomalous cooling dominates, by monitoring the estimated quantities-of-interest (QoIs) through a time-delayed regression analysis and find that the rapid decay in prompt LII signals is not accounted for by current evaporative mass loss model.

## Experimental setup

**Fig. 1:**
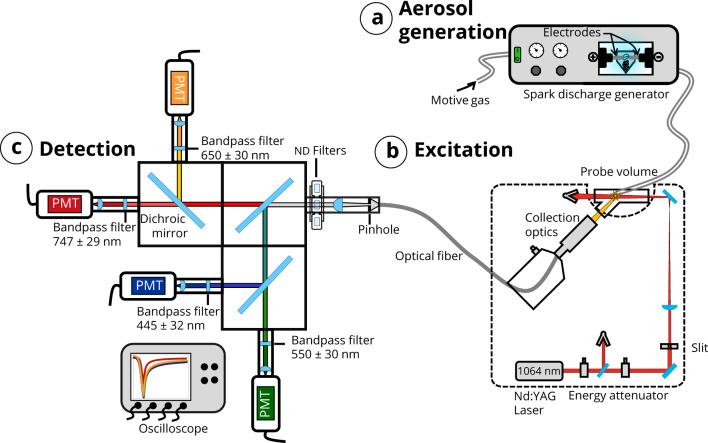
**a.** Aerosol generation, **b.** Excitation setup, **c.** Detection setup

**Fig. 2:**
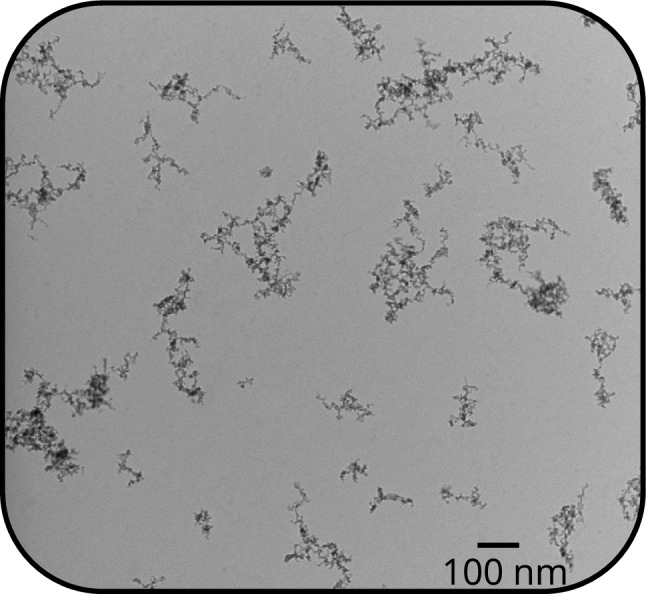
TEM micrograph of sampled spark-discharge generated iron nanoparticles

The iron nanoparticle aerosol was produced using a PALAS GFG spark discharge generator (Fig. 1a). Nitrogen gas was supplied at 1.2 bar to the generator, which was equipped with iron electrodes (ARMCO®, 99.85% iron) and operated at a spark frequency of 270 Hz. The iron nanoparticle aggregates produced by this generator have been described in detail in our previous work [14] with some example micrographs shown in Fig. 2. TEM analysis showed that the aggregates obey a lognormal size distribution with an average primary particle diameter of 6 nm and, on average, 126 primary particles per aggregate.

The aerosol is irradiated using a 1064 nm Nd:YAG laser operated at a repetition frequency of 20 Hz. The laser pulse has a Gaussian temporal profile with a pulse duration of ~ 8 ns FWHM. The laser beam passes through a variable attenuator consisting of two half-wave plates and a polarizer, then through a 2 × 1 mm^2^ rectangular ceramic aperture, followed by a convex lens, before finally entering the LII chamber through a window positioned at Brewster angle (Fig. 1b). Measurements are conducted at fluences between 1.8–3.14 mJ/mm^2^. The lower end of the fluence range used was dictated by the detection limit of our setup and the higher end by the maximum laser fluence achievable. Before laser irradiation, the spark-discharge-generated particles are assumed to be thermally equilibrated with the buffer gas at room temperature.

The detector arrangement is shown in Fig. 1c. Light emission from the heated aerosol is focused using two achromatic lenses with focal lengths of 250 and 100 mm, onto the entrance of an optical fiber (diameter: 1.5 mm, numerical aperture: 0.39, length: 2 m, Thorlabs). The fiber is connected to a multispectral detector system consisting of a series of dichroic mirrors and bandpass filters that decouple the broadband emission into four separate wavelength ranges (445 ± 32 nm, 550 ± 70 nm, 650 ± 70 nm, and 747 ± 29 nm), each mounted in front of four PMTs with rise times ranging from 0.57–0.78 ns. The respective signals were recorded every 0.4 ns with an oscilloscope (Teledyne Lecroy HDO6104, 1 GHz, 2.5 GS/s). The detector system was calibrated with a tungsten-halogen light source (Thorlabs, SLS202L) having a filament temperature of 2800 K, following the procedure described in Ref. [22] and taking into account the wavelength-dependent sensitivity at each detector channel. Each recorded LII signal is an average of 200 individual shots and a total of 30 averaged signals are collected.

## TiRe-LII measurement models

### TiRe-LII model

The temperature evolution, *T*(*t*), of a laser-heated aggregate, is modeled by the energy balance

1 $$ mc\frac{dT}{{dt}} = q_{{{\text{laser}}}} \left( t \right) - q_{{{\text{evap}}}} \left[ {T\left( t \right)} \right] - q_{{{\text{cond}}}} \left[ {T\left( t \right)} \right], $$

where *m* and *c* are the mass of the aggregate and the specific heat of the aggregate material, respectively, *q*_laser_(*t*) is the rate at which laser energy is absorbed by the aggregate, and *q*_evap_ and *q*_cond_ are the cooling rates by evaporation and heat conduction to the gas, respectively. These terms are articulated in Ref. [14] and its Supplementary Information.

The aggregate loses mass through evaporation according to

2 $$ \frac{dm}{{dt}} = - M_{{\text{v}}} \frac{{q_{{{\text{evap}}}} }}{{\Delta H_{{\text{v}}} }}, $$

where *M*_v_ and Δ*H*_v_ are the molar mass and latent heat of vaporization of the nanoparticle material, respectively; Δ*H*_v_ is computed using Watson’s equation [23]. The primary particle diameters change due to evaporative mass loss and temperature-dependent density variation according to *d*_p_(*T*_p_, *m*_p_) = [6*m*_p_/(π*ρ*_m_)]^1/3^, where the subscript “p” indicates isolated primary particles. In this work, the thermal accommodation coefficient is taken as 0.08 [9], and the mass accommodation coefficient, is taken to be unity as is commonly assumed in the literature for metal nanoparticles [6].

When primary particles are in point contact, the laser absorption model assumes that the particles absorb energy independently within the aggregate. Because the local EM field strength is influenced by the surrounding particles in an aggregate, each primary particle may in reality be exposed to different field strengths and thus be heated to different temperatures [21]. The energy absorbed by a non-sintered aggregate equals the sum of energy deposited in the individual particles. Because the metal aggregates are expected to absorb and emit outside of the Rayleigh limit, the local EM field is influenced by the aggregate structure and, consequently, the primary particles are expected to be at different temperatures at any instant [21].

After the onset of sintering (when primary particles are no longer in point contact but begin to overlap), the resulting aggregate is assumed to absorb laser energy as an electrically and thermally conductive structure, which is isothermal at any given instant. Because of the electrical contact between the particles, the absorbed energy depends on the cross-section of the entire aggregate. Consequently, the particle temperature depends on the compactness of the initial structure, as particles of different masses and thermal capacities may share a similar absorption cross-section [21]. When the aggregate structure changes upon coalescence, a spherical particle formed via coalescence from an aggregate would have absorbed more laser energy and would be hotter than a particle with the same diameter and mass that was compact throughout the laser interaction. This effect is discussed further in Sect. 4.2.

The evaporation model assumes that the particles within the aggregate remain in point contact and evaporate independently, which may not be accurate upon melting, sintering, and coalescence. The melting enthalpy is neglected since the energy associated with the solid-to-liquid phase change is orders of magnitude less than the laser heating (see Supplementary Information Sect. 1).

The spectral incandescence of an aerosol containing monodisperse aggregates is given by

3 $$ J_{\lambda } \left( t \right) = A_{\lambda } \Lambda {\mathbf{C}}_{{{\text{abs}},\uplambda }} \left( {{\mathbf{m}}_{\lambda } ,d_{{\text{p}}} ,{\mathbf{x}},N_{\text{p}} } \right) \cdot {\mathbf{I}}_{{\lambda ,\text{b}}} \left[ {{\mathbf{T}}\left( {d_{{\text{p}}} ,{\mathbf{x}},t} \right)} \right], $$

where *A*_λ_ and Λ are the calibration constant and the intensity scaling factor, respectively; *d*_p_ is the primary particle diameter, and **x**, **C**_abs,λ_, **I**_λb_, and **T** are vectors containing the centroid location, spectral absorption cross-section, blackbody spectral intensities, and temperatures of the primary particles, respectively, in the aggregate at time *t*. When all the aggregates coalesce into spheres, the incandescence signal is modeled as

4 $$ J_{\lambda } \left( t \right) = C_{\lambda } \Lambda \int_{0}^{\infty } {p\left( {d_{\text{p}}^{{\text{coalesc}}} } \right)C_{{{\text{abs}},\uplambda }} \left( {{\mathbf{m}}_{\lambda } ,d_{\text{p}}^{{\text{coalesc}}} } \right)I_{{\lambda ,{\text{b}}}} \left[ {T_{{\text{p}}} \left( {d_{\text{p}}^{{\text{coalesc}}} } \right)} \right]d\left( {d_{\text{p}}^{{\text{coalesc}}} } \right)} , $$

where *p*(*d*_p_^coalesc^) is the probability density function (PDF) that describes the diameters of the coalesced spheres. The width of this distribution originates mainly from the polydispersity of primary particle numbers, *p*(*N*_p_), in the original aggregate population. Finally, for a hypothetical monodisperse aerosol of spheres, the incandescent signal would be

5 $$ J_{\lambda } \left( t \right) = C_{\lambda } \Lambda C_{{{\text{abs}},\uplambda }} \left( {{\mathbf{m}}_{\lambda } ,d_{{\text{p}}} } \right)I_{{\lambda ,{\text{b}}}} \left[ {T_{{\text{p}}} \left( {d_{{\text{p}}} } \right)} \right]. $$

Often an effective temperature of the particle ensemble is approximated by computing a pyrometric temperature, which is most often done by assuming that the particles emit and absorb in the Rayleigh regime and *C*_abs,*λ*_ (**m**_*λ*_, *d*_p_) = (π^2^*d*_p_^3^/*λ*)*E*(**m**_*λ*_), where *E*(**m**_*λ*_) is the absorption function of the material [14]. Through this approximation, the pyrometric temperature *T*_p,pyro_ can be computed by a weighted nonlinear regression of the modeled spectral intensity to the measured spectral intensity. When two-color pyrometry is used to compute *T*_p,pyro_ from measured signals at any two detection wavelengths *λ*_1_ and *λ*_2_, this procedure can be done explicitly according to

6 $$ T_{{\text{p,pyro}}} = \frac{{hc_{0} }}{{k_{{\text{B}}} }}\left( {\frac{1}{{\lambda_{2} }} - \frac{1}{{\lambda_{1} }}} \right)\left[ {\ln \left( {\frac{{J_{\lambda ,1} }}{{J_{\lambda ,2} }}\frac{{E\left( {{\mathbf{m}}_{\lambda ,2} } \right)}}{{E\left( {{\mathbf{m}}_{\lambda ,1} } \right)}}\left( {\frac{{\lambda_{1} }}{{\lambda_{2} }}} \right)^{6} } \right)} \right]^{ - 1} , $$

where *c*_0_, *h*, and *k*_B_ are the speed of light, and the Planck and Boltzmann constants, respectively. For iron nanoparticles with diameters greater than ~ 3 nm, the refractive indices may be taken to be that of the bulk material (see Supplementary Information Sect. 2). In this work, we use ellipsometry data for bulk molten iron obtained by Shvarev et al*.* [24]. While the nanoparticles are solid for a short time after the onset of laser irradiation before melting, only the refractive index of molten iron is considered because the total signal is dominated by the hot molten particles as discussed further in Sect. 4.1.

Previous LII studies on metal nanoparticles often assumed the aerosol to consist of a population of isolated spheres corresponding to the primary particles of the aggregates, or a monodisperse aerosol. Properties were then inferred by regressing a spectroscopic model derived using Eqs. (4) or (5), respectively, while applying the Rayleigh approximation. However, this work accounts for the diameter of the coalesced sphere produced by the sintering of an aggregate containing *N*_p_ primary particles.

At any instant, the pyrometric temperature obtained using this procedure differs from the average sensible temperature of the particle ensemble within the probe volume for several reasons: (1) the particles are polydisperse in size, and therefore are expected to have different temperatures at any instant [13]; (2) the Rayleigh approximation does not generally hold for solid aggregates nor fully-sintered metal spheres [25–27]; (3) field enhancement effects within the aggregates during emission may further contribute to nonuniform temperatures [21]. Therefore, while *T*_p,pyro_ provides a rough indication of the instantaneous “average” temperature (although biased towards hotter particles [14]), it should not be interpreted as a true thermodynamic temperature. Moreover, since all these spectroscopic effects change as the particles are heated and melted, they may at least partially account for some of the heretofore unexplained transient phenomena in LII measurements. A further time-dependent artifact in the pyrometric temperature may arise from the fact that the hottest particles in an ensemble might cool faster due to evaporative cooling [21], even if the pyrometric temperature of the ensemble appears to be lower than the temperature for significant evaporative cooling.

### Modeling of aggregate sintering, coalescence, and absorption properties of metal nanoparticles

Our previous work showed that partial sintering enhances the absorption cross-sections of the aggregates, thereby raising the peak temperature compared to what would be expected from an aerosol of isolated spheres having diameters consistent with those of the primary particles in the initial aggregates [21]. We also hypothesized that apparent anomalous cooling could be explained, at least in part, by aggregates containing primary particles that are not fully sintered by the time that peak incandescence is reached. At this point, the aggregate structure would lead to localized field enhancements and, therefore, primary particles that may reach temperatures far above the median value. These particles would cool down through evaporation, subsequently manifesting in a rapid drop in the effective temperature decay rate [21]. However, the present work shows that the particles will have melted and transformed into spheres before the time of peak emission, so the previous hypothesis does not apply to the conditions investigated here (discussed further in Sect. 4.1). In this section, we present an overview of the model used to simulate aggregation, sintering and coalescing, and absorption cross-section of the resulting aggregate morphology.

Synthetic nanoparticle aggregate geometries are generated through the tunable cluster–cluster aggregation algorithm [28] with uniformly sized primary particles according to the fractal law *N*_p_ = *k*_f_ × (2*R*_g_/*d*_p_)^*D*f^, where *R*_g_, *k*_f_, and *D*_f_ are radius of gyration, fractal prefactor, and fractal dimension, respectively. Metal nanoparticles produced through spark discharge generation form aggregates through diffusion-limited aggregation [29] with representative fractal parameters *k*_f_ = 1.19 and *D*_f_ = 1.82 [30]. Analysis of TEM micrographs in our previous work showed primary particle diameters and number of particles per aggregate followed a distribution of ln*N*(6 nm, 1.3) and ln*N*(126, 1.77), respectively [14].

Viscous sintering commences once metal aggregates approach their melting point and is driven by the tendency of the aggregate to minimize its surface energy [31]. The characteristic time required for an aggregate to reform into a sphere via this process is [32]

7 $$ {\uptau } = \frac{{\eta r_{0} }}{\gamma }\left( {\frac{{N_{{\text{p}}} }}{2}} \right)^{{\left( {{1 \mathord{\left/ {\vphantom {1 {2 - {{D_{{\text{f}}} } \mathord{\left/ {\vphantom {{D_{{\text{f}}} } 6}} \right. \kern-0pt} 6}}}} \right. \kern-0pt} {2 - {{D_{{\text{f}}} } \mathord{\left/ {\vphantom {{D_{{\text{f}}} } 6}} \right. \kern-0pt} 6}}}} \right)}} , $$

where *η* and *γ* are the viscosity and surface tension of the material, respectively, and *r*_0_ is the initial radius of the primary particles within the aggregate. Since the coalescence of molten and liquid metals may be faster than the viscous sintering of solid metals as they approach their melting point, Eq. (7) is an upper limit of the time required for coalescence.

The geometric changes induced by the initial phase of sintering between two neighboring primary particles are represented by.

8 $$ C_{{\text{OV}}} = \frac{{r_{i} + r_{j} - d_{ij} }}{{r_{i} + r_{j} }}{; 0} \ge C_{{\text{OV}}} \ge 1, $$

where *C*_ov_ is an overlap factor, *r*_i_ and *r*_j_ are the radii of primary particles *i* and *j* respectively, and *d*_*ij*_ is the distance between their centers. The primary particle radii are increased by equal amounts to compensate for the overlapped volume.

The absorption efficiency of the aggregates is computed using the multi-sphere T-matrix (MSTM) method [33] for aggregates consisting of primary particles in point contact, the discrete dipole approximation (DDA) [34] for sintered aggregates, and Mie theory [25] for fully coalesced spheres. The T-matrix and DDA methods are implemented using MSTM version 3.0 [33] and DDSCAT 7.3 [34], respectively. These calculations demand special attention due to the large refractive index of metals. In the case of MSTM, a larger number of expansion terms are needed to reach convergence compared to what is expected for soot [35], while in the case of DDA, a small dipole spacing is needed. A convergence study showed that 20 expansion terms are needed for the MSTM computation and a mesh study determined that a 0.3 nm dipole spacing was sufficient for the DDA computation (see Supplementary Information Sect. 3). For the DDA calculations, 18 aggregate orientations spanning the full range of aggregate rotation were averaged to obtain the orientationally averaged *C*_abs,*λ*_. Results from vertically and horizontally polarized *E*-fields were also averaged. The biconjugate gradient method was used to iteratively solve for the dipole polarizabilities which were defined by the filtered coupled dipole method (recommended for large refractive index materials [34]). The ambient environment was taken to be air.

### Data regression

Deficiencies in the LII model may be elucidated from the residual between experimental incandescence traces and the corresponding best-fit simulations. In this work, the spectral incandescence data from all detection wavelengths are regressed through least squares minimization as described in Sect. 3.1. The free parameters are the particle diameter *d*_p_, the geometric standard deviation of an assumed log-normal particle-size distribution, *σ*, the TAC, *α*, and the peak temperature from which the particles cool, *T*_peak_. The LII signal is computed by evaluating Eqs. (1), (2), and (5). Regression to the incandescence signal, as opposed to the pyrometric temperatures derived through color pyrometry, avoids the error introduced by the Rayleigh approximation [10, 13].

## Results and discussion

### Analyzing the extent of sintering by the peak of the LII signal

**Fig. 3:**
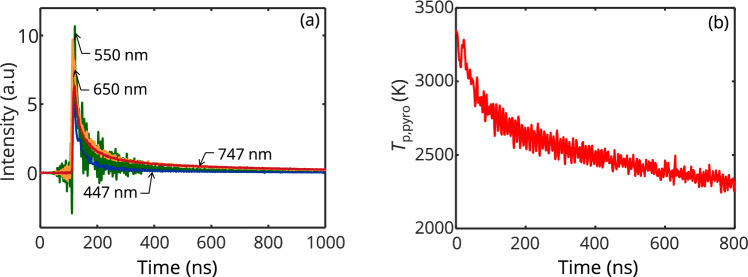
**a.** Measured LII signals at four detection wavelengths obtained from iron nanoparticles irradiated at a laser fluence of 2.7 mJ/mm^2^ **b.** Corresponding apparent pyrometric temperatures computed by a weighted nonlinear regression of the modeled spectral intensity (Eq. (5)) to the measured signals at four detection wavelengths centered at 445, 550, 650, and 747 nm. The pyrometric temperatures are estimated with *E*(**m**_λ_) determined from refractive indices obtained from ellipsometry [24]

Figure 3 shows a typical measured signal and the corresponding pyrometric temperature. While all channels in the detection setup are influenced by Q-switch noise and electronic noise from the surroundings, in our experiment the 550 nm was particularly noisy.

Since aggregates are expected to have extinction spectra that are distinct from isolated spheres [36], analyzing the spectral distribution of the peak LII signal may indicate the extent of sintering by the peak of the LII signal. This can be accomplished by comparing the spectral distribution at the LII peak, measured experimentally, with the spectral intensity distributions predicted for aggregates with varying degrees of sintering (*i.e.*, *C*_ov_ = 0%, 25%, 50%, 75%, and 100%) and containing 30, 50, 70, 100, 135, or 200 particles per aggregate. The particle diameter corresponding to 100% primary particle overlap is equivalent to the volume equivalent diameter of the aggregate according to *d*_100%_ = *d*_p_ *N*_p_^1/3^.

**Fig. 4:**
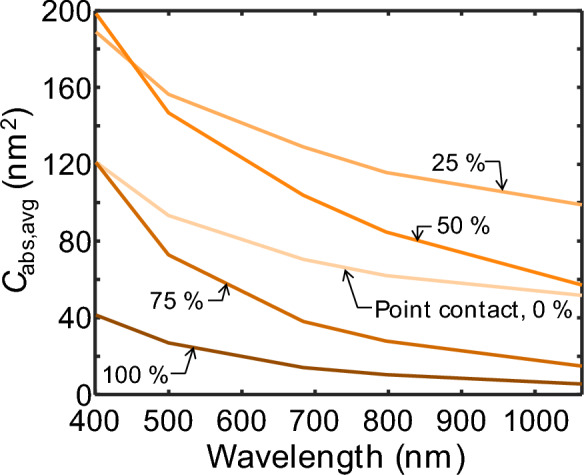
Average absorption cross-section of iron aggregates (*N*_p_ = 30, 50, 70, 100, 135, 200 drawn from ln*N*(126, 1.77)) computed from DDA at different sintering stages. The non-monotonic trend as the aggregate sinters from point contact is due to increasing dipole interaction at the sintering sites before reaching a maximum, after which *C*_abs,λ_ reduces due to the increasing structural compactness [21]

**Fig. 5:**
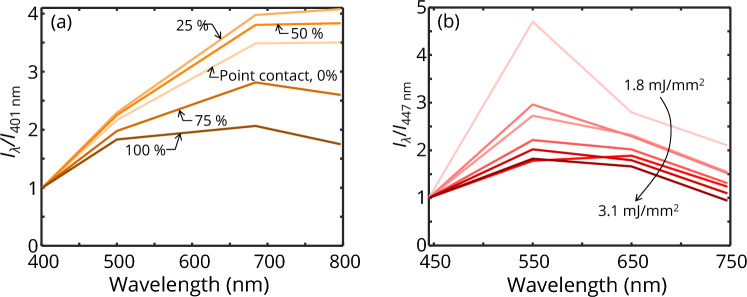
Spectral distribution of the LII emission at the time of the maximum LII signal **a.** Simulations for sintering stages with *C*_ov_ = 0%, 25%, 50%, 75%, and 100% which are all assumed to be at the same temperature of 3,300 K, **b.** Measured calibrated signal from iron nanoparticle aerosols. In all cases, emission intensities are normalized to the shortest emission wavelength

Modeling the spectrally-resolved peak LII emissions from the particle ensemble requires knowledge of the peak temperature reached by aggregates with different morphologies and varying degrees of sintering. Since this information is unavailable, it is temporarily assumed that the aggregates reach a uniform peak temperature of 3300 K, estimated by pyrometry (see Fig. 3). Then, the computed absorption cross-sections for each size class and sintering stage, computed from DDA at wavelengths of 401, 500, 684, and 797 nm, are weighted by the log-normal distribution, ln*N*(126, 1.77) to get an average absorption cross-section of the ensemble shown in Fig. 4. The peak of the incandescence signal at each sintering stage and each emission wavelength is then computed using Eq. (5) and normalized to the shortest emission wavelength as shown in Fig. 5a.

Figure 5 shows the simulated and measured spectral intensity at the peak incandescence for four detection channels. For the simulation results shown in Fig. 5a, the normalized spectral intensity increases with wavelength in accordance with the spectral absorption cross-section shown in Fig. 4. However, the spectral distribution of the signal depends on the degree of sintering (*i.e.*, varying *C*_ov_). Increasing *C*_ov_ up to 50% leads to a slight increase in intensity while maintaining the spectral shape. A further increase in *C*_ov_ leads to an overall decrease in signal intensity relative to the signal at the shortest wavelength, but is more pronounced at longer wavelengths, leading to a signal peak at around 700 nm. This trend is consistent with the magnitudes of the absorption cross-sections of each sintering stage shown in Fig. 4, with the fully coalesced aggregates (*C*_ov_ = 100%) having the lowest C_abs,797 nm_/C_ab,401 nm_ ratio and the 25% sintering stage having the highest. Figure 5b shows that the measured spectral shapes of the LII emission at all laser fluences have signal peaks in the visible range and are most consistent with simulated emission spectra for an aerosol of fully coalesced aggregates (*C*_ov_ = 100%). This observation suggests that the iron aggregates are coalesced, or nearly so, by the time the peak LII signal is reached, which is also consistent with observations of laser-irradiated samples of iron aggregates that show complete coalescence in TEM measurements [14]. Note that the measured spectral shape shown in Fig. 5b peaks around 550 nm, while the simulated data peaks around 700 nm. This difference may be attributed to the fact that the modeled peak incandescence is computed assuming that the particles are at the same peak temperature. However, we also note that the poor signal-to-noise ratio of the 550 nm detection channel (as observed in Fig. 3a) may also be a contributing factor.

Coalescence during laser heating is consistent with the outcome of Eq. (7), which suggests a characteristic viscous sintering (and thus coalescence) time of ~ 10^–2^ ns, much shorter than the duration of the laser pulse and the estimated measured heat-up time (time from the initial background signal to the peak of the LII signal) that ranges from 8 to 12 ns at all fluences. Since Eq. (7) only predicts the characteristic viscous sintering time, the metal aggregate may begin to sinter through a slower process such as grain-boundary diffusion [37] at lower temperatures. However, even the characteristic sintering time for grain-boundary sintering of iron nanoparticles above 800 K is already less than the laser pulse duration (see Supplementary Information Sect. 4.1). Note that the surface energy of the iron nanoparticles in the related size range depresses the melting point from 1811 K (bulk) to about 1483 K (see Supplementary Information Sect. 4.2). This further suggests that the particles are completely coalesced by the time the peak LII signal is reached. Hence, our hypothesis in Ref. [21] that suggests anomalous cooling may occur due to nonuniform heating and cooling of the primary particles within the aggregate as a result of the nonuniform laser absorption, could not apply to iron aggregates were the particles coalesce as rapidly as predicted by the viscous sintering model.

### Predicting the impact of coalescence on the LII signal

**Fig. 6:**
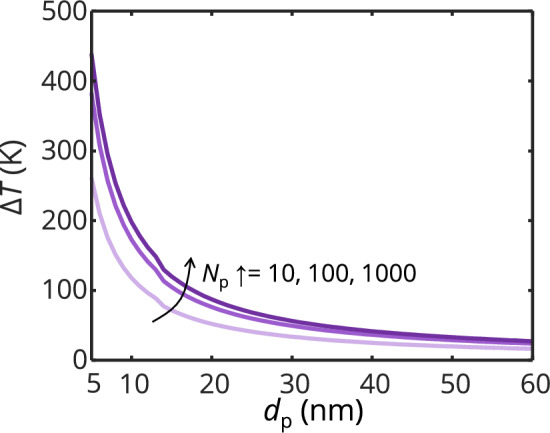
Temperature increase related to coalescence of aggregates as a function of primary particle size computed from the released surface free energy due to reduction in specific surface area

Sintering and coalescence are exothermic processes due to the release of surface free energy from the reduction in surface area (i.e.,* γ*Δ*A*_s_, where Δ*A*_s_ is the change of the surface area from an aggregate morphology to a sphere after coalescence). The temperature increase due to the energy gained from the reduction in surface area is

9 $$ Q_{{\text{SE}}} { = }m_{{\text{agg}}} c_{p} \Delta T. $$

where *Q*_SE_ is the surface energy gained through surface reduction, *m*_agg_ is the mass of the aggregate, *c*_*p*_ is the specific heat, and Δ*T* is the equivalent temperature increase. From the determined released energy due to coalescence (See Supplementary Information Sect. 4.3), for aggregates with *N*_p_ = 10, 100, 1000, the temperature increase as a function of primary particle diameters (with melting point depression considered) is shown in Fig. 6. For an aggregate consisting of primary particles having a diameter of 6 nm, the equivalent temperature increase is ~ 300 K.

The temperature evolution of laser-heated iron aggregates is modeled as follows. In the initial particle heating phase (*i.e.*, before sintering), the solid aggregates are modeled as being in point contact. Once the melting temperature is reached, the aggregates coalesce immediately into spheres, which is justified by the very short coalescence time predicted by Eq. (7), and the surface energy released according to Eq. (9) is added to the sensible energy of the sphere. The resulting temperature, *T*_melt_ + Δ*T*, is then used as an initial particle temperature to calculate the further heat-up by the remainder of the laser pulse, while using the optical properties of the liquid sphere.

**Fig. 7:**
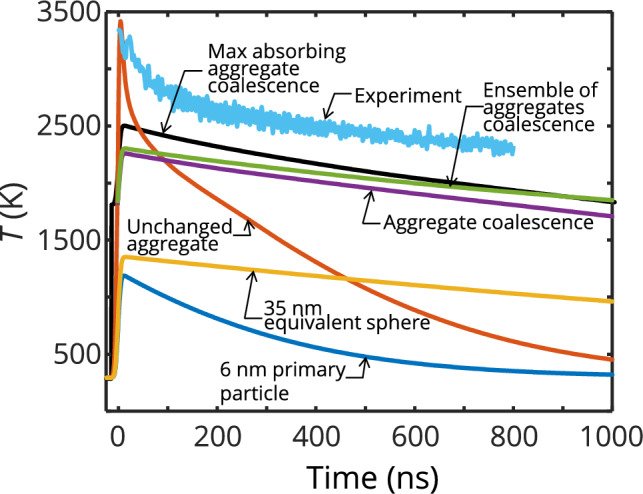
Simulated temperature profiles after laser heating for several conditions: A 6-nm diameter primary particle (dark blue), an aggregate unaffected by sintering (200 primary particles of 6 nm in diameter in point contact, red), and an equivalent sphere (35 nm) of the aggregate (yellow). Three scenarios following the step model transitioning from aggregate to sphere: A single aggregate in point contact (purple), an ensemble of aggregates in point contact (green), and a single partially sintered aggregate (black). All simulations are compared to the measured experimental temperature. A mid-range fluence of about 2.7 mJ/mm^2^ in the range of fluences used during experiments was used in both simulations and the experiment shown here

Figure 7 compares different hypothetical scenarios for the modeled particle temperature against the measured effective temperature (light blue) for a laser fluence of 2.7 mJ/mm^2^. The two extreme cases are represented by individual 6-nm primary particles and low *D*_f_ aggregates in point contact from 200 primary particles that are assumed to maintain their original structure. The latter case reaches the highest heat-up temperature because it maintains a large optical cross-section throughout laser heating. Both structures show fast cooling due to their large surface-to-volume ratio.

The purple curve follows the step model described above, where the particle coalesces into a 35-nm diameter sphere upon melting. Since this occurs early in the laser pulse, the peak temperature is far lower than in the previous cases because of the strongly reduced absorption cross-section upon coalescence. It is, however, significantly higher compared to the scenario in which the particle starts as a 35-nm diameter sphere, since, in the former scenario the enhanced absorption cross-section of the aggregate prior to melting, combined with the surface energy released during melting, augments the peak temperature by ~ 900 K. In both cases, the temperature decay is significantly slower compared to the blue and red cases because of the lower surface-to-volume ratio. In other words, a particle that coalesces during the laser pulse changes its interaction cross-section between laser absorption during heating and emission during cooling and thus would be contributing to the observed apparent “excessive absorption” since the sintered sphere would reach higher temperatures compared to an identically sized sphere with an unchanged morphology during the LII process.

The above scenarios pertain to individual particles and should not be compared directly to the effective temperature, which arises from contributions of all size classes. The green curve in Fig. 7 shows the modeled effective temperature for an ensemble of aggregates having primary particle diameters of 6 nm and an *N*_p_ distribution of ln*N*(200, 1.8). The aggregates transform into equivalent spheres upon melting according to the step-model described above. The solid aggregates are assumed to absorb light as would an aggregate containing 200 primary particles prior to melting, for computational expediency. However, the temperature enhancement due to absorbed surface energy and the absorption cross-section of the coalesced spheres are calculated according to the correct size classes. The effective temperature of the ensemble of polydisperse aggregates is higher than that of a monodisperse ensemble due to the fact that the hottest particles within the ensemble have a disproportionately large influence on the effective temperature [14]. The resulting peak temperature, however, is still below the experimentally determined pyrometric temperature.

Our previous work showed that partially sintered aggregates have larger absorption cross-sections compared to point-contact aggregates [21]. While the DDA method can be used to estimated the absorption cross-sections of partially sintered aggregates, the estimates are best used in a comparative capacity (as opposed to using the estimated absolute values) because of the error associated with DDA for large refractive index materials. Instead, we assume a hypothetically maximally absorbing aggregate (with a specific absorption cross-section about five times larger than that of the point-contact aggregate, resulting in a vertical increase in temperature prior to coalescence) that coalesces upon melting. According to the step model described above, this leads to an earlier time of coalescence and thus a larger fraction of the laser pulse interacts with the equivalent sphere instead of the aggregate. The combined effect leads to an increase of the predicted peak temperature by ~ 250 K temperature compared to the point-contact aggregates (Fig. 7, black). However, even this increased temperature remains below the apparent temperature derived from the measurement. Actual partially sintered aggregates would be expected to have temperatures between the maximally absorbing aggregate and the point-contact aggregate.

The peak temperature of the aggregate in point-contact (red) is comparable to the experimentally determined pyrometric temperature but this assumes that the aggregate structure remains unchanged, which is unlikely since the particles reach temperatures that exceed the melting point of the primary particles. Compared to the experimental data, the sintered structure cools down too slowly, while the non-sintered is too fast, which might indicate that in reality, a combination of such structures is present. We note here that while particles in the probe volume are expected to equilibrate with the ~ 300 K bath gas at later cooling times, their slower cooling—due to higher heat capacity and lower surface-to-volume ratio compared to 6 nm primary particles—is consistent with model predictions in Fig. 11, where large particles (150 and 250 nm) remain at high temperatures when cooling from the estimated peak pyrometric temperatures.

Note that the aggregate is modeled to absorb laser energy uniformly and absorption non-uniformities within the aggregate are ignored since sintering will lead to an isothermal aggregate. In addition, we note here the MSTM-estimated absorption cross-section is obtained with a larger number of expansion terms (to ensure convergence) than was used in our previous work [21] which previously underestimated the true absorption cross-section (see Supplementary Information Sect. 3).

Assuming sudden coalescence from an aggregate structure to a sphere upon melting, may excessively simplify the process while also greatly reducing the absorbed laser energy resulting in lower peak temperatures as seen in Fig. 7. There is currently no consolidated model to investigate the time-resolved aggregate sintering on the LII signal and temperature.

### Analysis of the pyrometrically-determined peak temperatures

Until now, the heating phase of the LII signal has been analyzed to elucidate the laser–nanoparticle interaction, however, it is the cool-down phase of the particles and the associated LII signal that provides information about the particle size distribution and other QoIs. The temporal variation of the LII signal during the cool-down phase can be conceptually divided into prompt and delayed portions; the prompt signal extends from the peak signal to ~ 50 ns following the peak, which is followed by the delayed signal. The apparent anomalous signal behavior mostly influences the prompt phase [19] but has been observed to last for as long as 400 ns in the case of LII measurements on molybdenum aggregates [9].

**Fig. 8:**
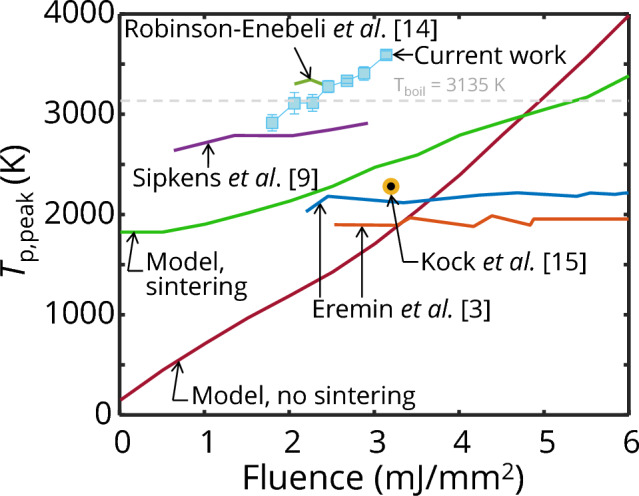
Apparent particle temperatures derived from LII signals as a function of laser fluence, compared with simulated temperatures for iron nanoparticles with a diameter distribution of ln*N*(56 nm, 2) obtained from Eqs. (1), (5), and (6) (model, no sintering) and simulated temperatures from an aggregate size distribution of ln*N*(200, 1.8) with sintering and surface energy release considered (Model, sintering). Peak temperatures from the literature are obtained from Refs. [3, 9, 14, 15] and re-evaluated with the *E*(**m**_λ_) determined from refractive indices obtained from ellipsometry [24]

Figure 8 compares LII fluence curves for iron nanoparticle aggregates reported in the literature with those obtained from the current experimental data. Note that the morphologies of the iron aggregates from the earlier studies all differ from each other and this work. An expected fluence curve can be simulated from an initial aggregate distribution of *N*_p_ = ln*N*(200, 1.8) (informed by TEM analysis) while also considering the surface energy released during coalescence. This simulation is also compared to the fluence curve based on the diameters of coalesced aggregates, ~ ln*N*(56 nm, 2), based on iron aggregates captured on a TEM grid and then heated using the laser pulse [14]. The coalesced particles within the aerosol have different peak temperatures and the emitted incandescence signals are integrated, according to Eq. (4), and subsequently used to determine simulated pyrometric temperatures. Unlike soot, Fig. 8 shows that the simulated fluence curves do not exhibit a plateau temperature in the fluence range typical of LII experiments [38] (the plateau region occurs at much higher fluences, see Supplementary Information Sect. 5). The peak pyrometric temperatures obtained from the current measured data are higher than the simulated value. This may be due to the actual size distribution of the coalesced particles within the probe volume being different from the estimated size distribution of the irradiated TEM sampled iron aggregates.

The prompt LII signals could potentially be corrupted by non-incandescent emissions that could bias the inferred peak temperatures. These emissions may originate from thermal or laser-induced emission of atomic or molecular gas-phase species [39], recombinative chemiluminescence [19], or plasma emission [20, 40]. These signals can differ in temporal characteristics depending on excited state lifetimes and formation kinetics of the related excited species. Vander Wal et al*.* [19] experimentally investigated the interference of non-incandescent emissions by analyzing both spectrally- and time-resolved data based on the assumption that LII and the potential interference have highly distinct fluence dependences and temporal characteristics so that significant non-LII interference would result in a temporal variation of the total signal as a function of laser fluence. As we do not observe such changes, it seems unlikely that the signals were affected by appreciable non-incandescent emission (see Supplementary Information Sect. 6).

### Quantities-of-interest inference from prompt and delayed LII signals

Given the uncertainty attached to the prompt signal, it is common practice to exclude this data when inferring quantities-of-interest like the size distribution parameters from the LII data, up to a certain delay time after the peak incandescence signal [10, 41]. In this section, we explore how increasing this delay time impacts parameters inferred from the signal decays. This method of time-delayed detection has also been performed for metal nanoparticle LII [19].

The LII model used to analyze the data assumes that the aerosol consists of isolated molten iron spheres. Only the cool-down phase of the LII signal is included in the regression because, as established in Sects. 4.1 and 4.2, the physical understanding of the heating phase for iron nanoparticles is incomplete (see also Supplementary Information Sect.  7 for attempts to include the heating phase in the regression). Accordingly, this treatment imposes a uniform particle temperature equal to the pyrometric temperature inferred from the peak LII signal. However, while this may be reasonable for aerosols of soot particles that absorb and emit in the Rayleigh regime, in the case of metal nanoparticles it introduces an unavoidable, and potentially substantial, model error into the analysis since particles of different sizes and morphologies absorb laser energy and emit incandescence in a manner that is not proportional to their volume [13], and consequently, the peak particle temperature is expected to be far from uniform.

**Fig. 9:**
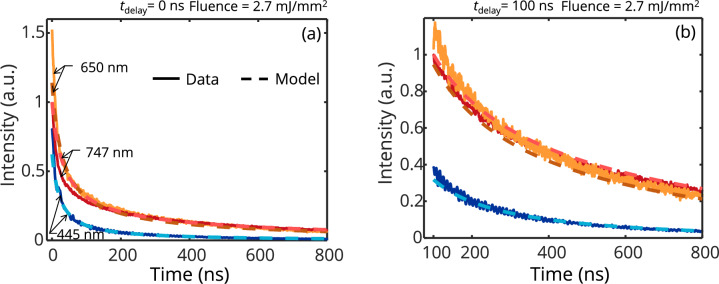
Regression of the LII model (Eqs. (1) and (4)) to the data at a *t*_delay_ of **a.** 0 ns and **b.** 100 ns

A total of 30 signals were obtained, each averaged over 200 shots. A weighted nonlinear regression is used to infer two combinations of four QoIs that include the mean particle diameter, *d*_p_, the geometric standard deviation of the distribution, *σ*, the thermal accommodation coefficient, *α*, and the model peak temperature, *T*_peak_. Case I has the QoI combination: [*d*_p_, *σ*, *α*, *T*_peak_] and Case II assumes a monodisperse aerosol with the combination: [*d*_p_, *α*, *T*_peak_]. The regression is performed at 450, 650, and 747 nm, avoiding the noisy 550 nm detection channel, and at different time delays, *t*_delay_, after peak incandescence. Figure 9 shows that the LII modeled data is in better agreement with the experimental data at a longer time delay, *t*_delay_, suggesting that, as expected, the model cannot accurately capture the prompt signals.

**Fig. 10:**
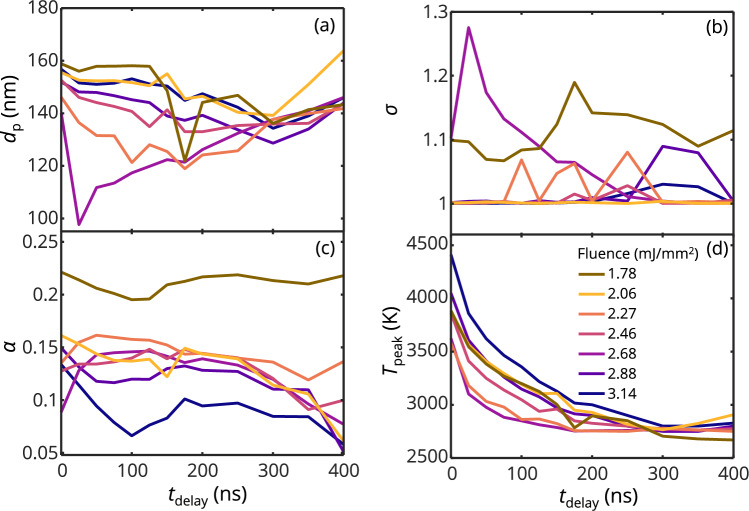
Inferred QoIs from Case I including, **a.** *d*_p,_ **b.** *σ*, **c.** *α*, and **d.** *T*_peak_ as a function of *t*_delay_ at difference laser fluences. Inferrences from the lowest fluence may be unreliable due to the large noise levels in the data

The inferred QoI from Case I are shown in Fig. 10. The inferred geometric mean particle diameter ranges from about 100–160 nm at shorter *t*_delay_, and this range narrows with increasing *t*_delay_ to 140–150 nm at the longest time delay of 400 ns. Because the inference procedure imposes a uniform initial temperature for all particle sizes, the inferred size distributions are uniform or nearly uniform (*σ* ≈ 1). While the true size distribution of coalesced spheres within the aerosol is unknown, our previous examination of iron aggregates deposited TEM grid and irradiated with a laser pulse suggests that the aggregates coalesce to a final size distribution of *d*_p_^coalesc^ ≈ ln*N*(56 nm, 2), which is inconsistent with the inferred size distribution. The TEM-estimated final size distribution of the coalesced particles was also inconsistent with the expected equivalent diameter of ~ 35 nm produced from the aggregate distribution of *p*(*N*_p_) = ln*N*(126, 1.77) assuming a uniform primary particle diameter of 6 nm. The reason for this discrepancy remains unknown, although, in the case of the particles irradiated on the TEM grid, the coalesced particles may consist of several “merged” aggregates.

**Fig. 11:**
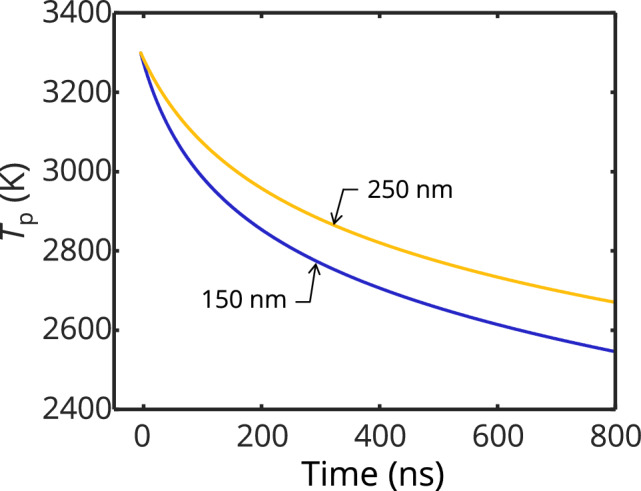
Comparison of the rate of evaporative cooling for iron nanoparticles with diameters of 150 and 250 nm, cooling from the same peak temperature of 3300 K

The inferred diameters are expected to be biased towards hotter particles since these particles dominate the emitted incandescence. In our previous work, it was determined that the particles with the highest peak temperatures had diameters of approximately 150 and 250 nm [14]; smaller particles have a lower absorption cross-section, while the temperatures of the larger particles are limited by their thermal mass. In this work, the inferred diameters are more in line with the lowest end of this range. Figure 11 shows that for the same peak temperature, the evaporative cooling rate for a 150-nm diameter particle is higher than that of a 250-nm diameter particle. In addition, the inferred model peak temperatures, particularly at shorter time delays, are higher than the effective pyrometric temperatures. Hence, the regression tends toward size classes that are more likely to produce the highest temperatures while also having the fastest evaporative cooling following the prompt signal.

Excluding the earlier portions of the signals increases the influence of polydispersity on the temperature distributions at later cooling times. Also, while the inferred TACs are “effective” due to the temperature distribution within the probe volume from different size classes, they are consistent with the values reported in the literature (0.05–0.1 [9]). Inferred peak temperatures are also higher than the measured pyrometric temperatures of ~ 3300 K at a lower *t*_delay_ but converge to lower temperatures at a longer *t*_delay_, however, compared to the pyrometric temperature shown in Fig. 3b the temperatures remain higher at longer cooling times. Higher temperatures are inferred compared to the pyrometric temperatures due to the use of the more accurate Mie absorption cross-sections that have larger *C*_abs,*λ*2_/*C*_abs,*λ*1_ ratio compared to the Rayleigh approximations which have a strong influence on the approximation of the pyrometric temperature. The reduction of the *T*_peak_ at a longer *t*_delay_ is expected since the temperatures are lower with the removal of more of the prompt signals. The narrowing distribution from all fluences with increasing *t*_delay_ indicate phenomena close to the peak signal that are not captured by the model.

**Fig. 12:**
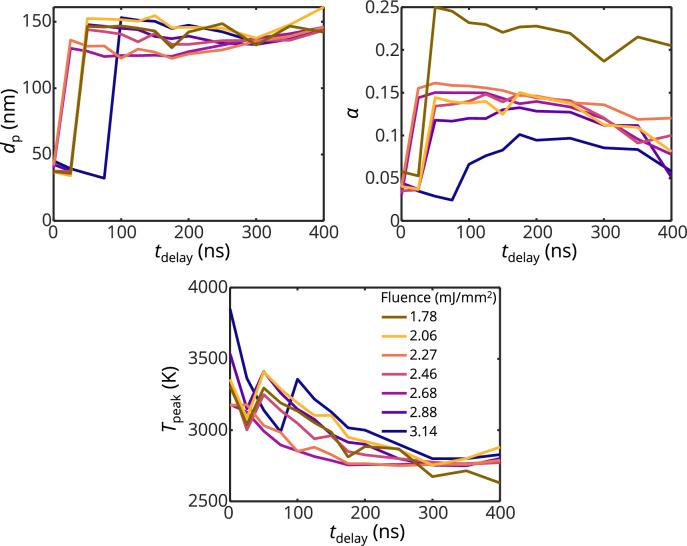
Inferred QoIs from Case II including, **a.** *d*_p,_ **b.** *σ*, **c.** *T*_peak_ as a function of *t*_delay_ at difference laser fluences

Case II results are shown in Fig. 12. Inferred QoIs at *t*_delay_ > 100 ns are generally consistent with trends observed for Case I. At *t*_delay_ < 100 ns, the inferred diameters and TACs are much lower including the goodness-of-fit. It is not clear why smaller diameters and TACs are inferred at *t*_delay_ < 100 ns, however, this causes lower model peak temperatures to be inferred with prompt signals compared to Case I which eventually increase at longer time delays when the inferred diameters are consistent with Case I.

**Fig. 13:**
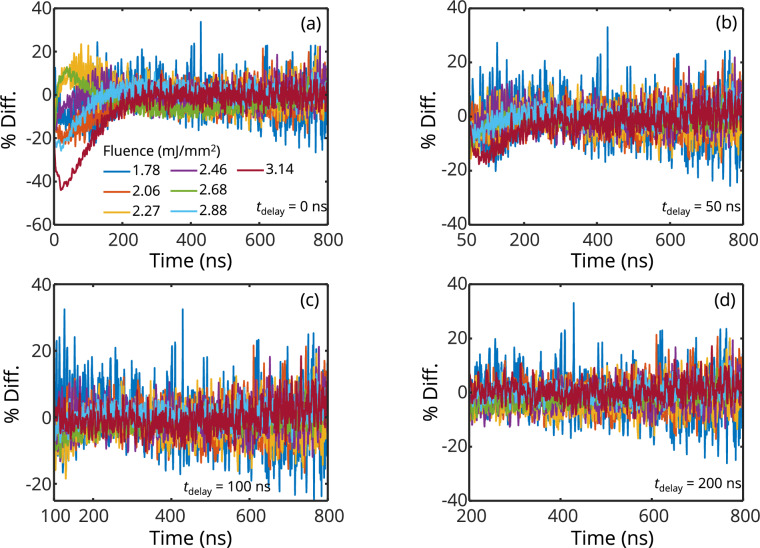
Residuals of the LII model regression to the emitted signals for different laser fluence at time delays of **a.** 0 ns **b.** 50 ns **c.** 100 ns, and, **d.** 200 ns. For each fluence, the residuals are averaged from those at the detection wavelength of 445, 650, and 747 nm

Some insight into model deficiencies may be gained from the residual between the measured intensity and the best-fit model. Figure 13 shows that, at *t*_delay_ = 0 ns, the residuals at all fluences reduce and converge to zero at *t* = 200 ns, after which the model slightly underestimates the data for some fluences. By *t*_delay_ = 100 ns, all residuals are unbiased for the remaining duration of the LII signal, but this does not mean that the corresponding inferred QoIs are accurate. Figure 13 also shows that the evaporation model does not capture the prompt signal decay, with a percent difference of as much as 40% at the highest fluence of 3.14 mJ/mm^2^. In this respect, the assumption of uniform peak particle temperatures may be a leading source of error, which can be eliminated with modifications (such as accurately accounting for time-resolved sintering). An experimental measure could be taken to reduce the contribution from the rapid unexplained cooling during the prompt signal by avoiding fluences that induce evaporation.

## Conclusions

While it is normally used to measure soot, time-resolved laser-induced incandescence (TiRe-LII) is increasingly applied to other materials, including metal aggregates. However, especially in the case of metal particles, current LII models cannot explain commonly observed features in the signals, particularly the observed peak pyrometric temperature is higher than expected for a given laser fluence, and the particle temperatures appear to decay faster than can be explained using conventional heat transfer models.

This work shows that laser-irradiated iron aggregates are likely to coalesce during the laser pulse once reaching their melting point, producing an aerosol of spheres having diameters determined by the total mass of the original aggregate. This affects the particle temperatures in a number of ways: (1) metal nanoparticle aggregates have significantly larger specific (mass-normalized) absorption cross-sections compared to spheres, which influences the absorbed laser energy per mass; (2) the surface energy of the aggregate is transferred to the sensible energy of the particle upon coalescence, thereby increasing its temperature; (3) the reduced surface-to-volume ratio reduces heat transfer to the surrounding gas, and (4) the absorption cross-section evolves as an aggregate in point contact sinters in a manner that depends on wavelength and likely reduces field-enhancements within the aggregate due to increased electrical contact. (5) Upon coalescence, particle ensembles with initial variations in aggregate size lead to ensembles of similarly-sized spherical particles with strongly varying temperatures, where the apparent temperature is increased because the pyrometric temperature measurement is biased towards the high-temperature part of the distribution.

Based on simulations of viscous sintering and coalescence times, we derive that coalescence occurs almost instantaneously during laser heating once the melting temperature has been reached. As a consequence, during the laser pulse, the specific absorption cross-section changes significantly, reducing laser heating. At the same time, the coalescence results in the release of surface energy, increasing the particle temperature. According to our evaluation, the loss in absorbed laser energy due to the reduction in absorption cross-section is far larger than the increase in sensible energy from the reduced specific surface area. Compared to the particle size after laser heating and coalescence, the particles have gained additional energy through enhanced absorption in the initial phase and through conversion of surface energy, thus contributing to the effect of “excessive absorption” described before for metal particles. Nevertheless, the current model can not yet fully explain the peak temperatures and temperature decayes observed experimentally. We discount the possibility that the unexpectedly high peak temperatures could be due to signal contamination by non-incandescent emissions in the fluence range applied in our measurements.

A further complication of the particle coalescence is that the particle peak temperatures are expected to be highly nonuniform in the particle ensemble. Since there is presently no model that adequately explains the details of laser-heating process during the structural change of the particles, we instead analyze the cooling phase of the signal by assuming that all the particles reach a common peak temperature. Analyses were started at varying delay times after the peak LII signal. The analysis showed that the current LII evaporation model does not accurately capture the prompt LII signals, however, the delayed signals are well captured by the conduction model. This may be a result of the assumption of uniform temperature, which is most likely not the case within the probe volume. Consequently, the inferred quantities-of-interest may not be representative of the actual particle diameters after coalescence in the aerosol, rather, they are diameters that most explain the high peak temperatures and rapid rate of evaporation.

Further experiments will be carried out with high melting and boiling point materials, which will provide complementary results for the current analysis including (i) slower sintering, resulting in incomplete sintering by the LII peak, providing an opportunity to compare the spectral emission at the peak of the LII signal to that of a fully sintered particle, and (ii) high boiling point resulting in slower cooling rates that may be dominated by conduction cooling, hence avoiding the inclusion of the evaporation model and possibly the absence of the anomalous cooling phenomenon. We will also investigate iron nanoparticles having different degrees of aggregation since the hypothesized effects of morphology on the absorption cross-section and specific surface energy depend strongly on the primary particle diameter, number of primary particles per aggregate, and aggregate structure.

## Supplementary Information

Below is the link to the electronic supplementary material.

Supplementary file1 (DOCX 3015 KB)

## Data Availability

No datasets were generated or analysed during the current study.

## Declarations

### Data transparency

The datasets measured and generated during and/or analyzed during the current study are available from the corresponding author on reasonable request.
