# Supplementary material for: Laser-induced incandescence of iron nanoparticles: effects of laser-induced sintering and coalescence
Source: Appl Phys B. 2025 Jun 11;131(7):135. doi: 10.1007/s00340-025-08504-0 (PMC12159127; doi:10.1007/s00340-025-08504-0)
Supplement: Supplementary file 1 — Supplementary file1 (DOCX 3015 KB) [file 340_2025_8504_MOESM1_ESM.docx]

**Laser-induced incandescence of iron nanoparticles:
Effects of laser-induced sintering and coalescence**

Stephen Robinson-Enebeli^1,2*^, Christof Schulz^2^, Kyle J Daun^1^

^1^Department of Mechanical and Mechatronics Engineering, University of Waterloo, 200 University Ave W, Waterloo, ON, Canada

^2^EMPI, Institute for Energy and Materials Processes – Reactive Fluids, and CENIDE, Center for Nanointegration Duisburg Essen, University of Duisburg-Essen, 47048, Duisburg, Germany

**Supplementary information**

1. **Melting model**

As opposed to soot particles that mostly sublime before melting at temperatures in excess of 4450 K [1], most metal nanoparticles melt long before reaching their boiling point. Filippov *et al.* [2] note this and model melting with an expression similar to that of evaporation

$$q_{\text{melt}}\left(T_{\text{p}},d_{\text{p}}\right)=\frac{\Delta H_{\text{m}}\left(T_{\text{p}}\right)}{M_{v}}\frac{dm_{\text{p,m}}}{dt}$$

where ∆*H*_m_(*T*_p_) is the temperature-dependent molar enthalpy of melting, *M*_v_ is the molar mass of the evaporated material, where the subscript v stands for vapor and *dm*_p,m_/*dt* is the rate of mass conversion from solid to liquid state (*i.e.*, the rate of phase transition). However, they do not provide an expression for *dm*_p,m_/*dt*; instead, the temperature of the nanoparticle is held constant until enough energy has been absorbed by (during heating) or removed from (during cooling) the nanoparticle, similar to the treatment by Michelsen [1]. To determine a conservative estimate of the total energy required to completely melt the aggregate structure, Eq. can be simplified to

$q_{\text{melt}}\left(T_{\text{p}},d_{\text{p}}\right)=\frac{\Delta H_{\text{m}}\left(T_{\text{p}}\right)}{M_{\text{v}}}m_{tot}$,

where *m*_tot_ is the total mass of the aggregate before laser irradiation. Using the thermophysical properties: *M*_v_ = 55.85 $\times$ 10^–3^ kg/mol and ∆*H*_m_ = 13.8 $\times$ 10^3^ J/mol [3], the required energy is in the order of 10^–14^ J which is much less than the amount of energy deposited by the laser (10^–10^ J) within the first 1 ns. However, as determined by Figure 1, melting temperatures are not reached until a significant amount of laser energy has been deposited. This shows the highly dynamic nature of the irradiation process, *i.e.*, the energy required to melt the structure has been instantaneously deposited but phase change and temperature increase require a finite time to take place which ideally is described by *dm*_p,m_/*dt* in Eq. . However, no reported measurement data has shown observable features of such phase transitions during LII experiments.

**Figure 1:** Temporal temperature variation of a laser-irradiated iron aggregate (N_p_ = 135) at 2 mJ/mm^2^ compared to the absorbed laser energy. The change in morphology upon melting is not considered here.

1. **Refractive index of small particles**

The computed temperatures depend on the spectral refractive index **m***_λ_* that is also used to compute *E*(**m***_λ_*). The primary particles within the spark discharge-generated aggregates are ~6nm in diameter which may result in optical properties that differ from the bulk. At particle diameters smaller than 10 nm, the refractive index of the material may differ from the bulk due to changes in atomic structure or particle surface (caused by changes in electron localization or coordination number) [4]. To estimate the expected change for iron nanoparticles, a modified Drude model [4] is used to account for the increased collisions at the particle boundary

$$\varepsilon_{dp}=\varepsilon_{b}+\omega_{p}^{2}\left[\frac{1}{\omega^{2}+\Gamma_{\infty}^{2}}-\frac{1}{\omega^{2}+\Gamma_{dp}^{2}}\right]+i\omega_{p}^{2}\left[\frac{\Gamma_{dp}}{\omega\left(\omega^{2}+\Gamma_{dp}^{2}\right)}-\frac{\Gamma_{\infty}}{\omega\left(\omega^{2}+\Gamma_{\infty}^{2}\right)}\right]$$

Where *ε*_dp_ is the permittivity of the particle, a frequency, *ω*, modified from the permittivity of the bulk material *ε*_b_, *ω*_p_ and *Γ*_∞_ are the plasma and relaxation frequency of the bulk respectively, and *Γ*_dp_ = 2*Av*_F_/*d*_p_, is the relaxation frequency within the particle, *A* is a theory-dependent property on the order of 1 and *v*_F_ is the Fermi velocity. Values of *ω*_p_ and *Γ*_∞_ are obtained from Ref. [5]. The permittivity of bulk iron is obtained from the refractive indices of bulk molten iron derived from ellipsometry [6]. Figure 2 shows that, for iron nanoparticles with diameters greater than ~3 nm, the refractive indices do not differ significantly from that of the bulk, hence, in this work, we assume the aggregate is molten upon laser irradiation and use measured data from ellipsometry for bulk molten iron obtained by Shvarev *et al.* [6].

**Figure 2: a.** Size-dependent permittivity of iron nanoparticles computed from Eq. (9)
**b.** Corresponding absorption function. Data for particle diameters of 1, 3, 5, and 10 nm and bulk are shown.

1. **Convergence and mesh study**
   1. **Multisphere T-matrix method (MSTM)**

When using the multisphere T-matrix method (MSTM) in the case of two touching spheres, with a size parameter $\ll$ 1 and a large refractive index, it takes a large number of expansion orders to resolve the vector spherical harmonic expansion and reach convergence, *i.e.*, the inclusion of higher order terms [7] compared to, for example, soot that may require much less. Figure 3 shows the number of higher-order terms needed to reach convergence when computing the absorption efficiency of a 70-primary particle iron aggregate irradiated at 1064 nm.

**Figure 3:** MSTM computed absorption efficiency vs. number of orders of expansion required in the vector spherical harmonic expansion for a 70 primary-particle liquid iron aggregate irradiated at 1064 nm.

As seen from Figure 3, results of *Q*_abs_ converge after about ~30 orders of expansion have been included. The exact number of expansion orders to reach convergence may vary depending on aggregate size and index of refraction. To strike a balance between computation effort and accuracy, we choose to include 20 orders of magnitude in our calculations which results in ~10 % difference relative to the highly converged result.

- 1. **Discrete dipole approximation (DDA)**

In addition to the requirement that the dipole spacing used in the discrete dipole approximation (DDA) calculation should be smaller than any structural features of the target structure, the dipole spacing should also follow the criteria: |**m**|*kd* << 1, where **m** is the refractive index of the material, *k* = 2π/*λ* is the wavenumber, and *d* is the dipole spacing. These criteria are valid when |**m** **–** 1| ≲ 3, however, when the refractive index is large, as in the case of metals and the magnitude of the refractive index falls outside this range, much smaller dipole spacings are needed [8]. The DDA method tends to overestimate the absorption cross-section outside the valid range. Figure 4, shows the evolution of the accuracy of DDA at different dipole spacings relative to the full Mie theory for a 6-nm diameter molten iron sphere.

**Figure 4:** Comparing the accuracy of the DDA method to that of Mie theory for a molten iron sphere with a diameter of 6 nm irradiated at 1064 nm. The refractive index of molten iron are used according to Ref. [6].

Significant computational efforts are required at smaller dipole spacings. For example, at a dipole spacing of 0.35 nm, the computation is completed in two minutes compared to three hours at a dipole spacing of 0.025 nm. We choose a dipole spacing of about 0.3 nm, with a corresponding error of about 18 % according to Figure 4. Other methods to compute light interactions with matter, such as the finite element method, have shown promise in producing more accurate results than DDA when investigating materials with high refractive indices [9].

1. **Sintering and melting temperature of the nanoparticles**
   1. **Grain-boundary sintering**

Below the melting temperature of a crystalline material, sintering occurs through grain boundary diffusion [10,11]. The characteristic grain boundary sintering time, *τ*_gb_, of two equally sized nanoparticles is estimated according to [11]

$$\tau_{gb}=\frac{k_{B}Tr_{p,0}^{4}}{2\pi D_{v}C_{v0}\delta_{gb}\gamma\Omega}$$

where *r*_p,0_ is the initial radius of the particles, *k*_B_ is the Boltzmann constant, *T* is the temperature of the particle, *γ* is the surface tension, *δ*_gb_ is the grain boundary thickness, *Ω* is the volume of the atomic vacancy, *C*_v0_ initial fraction of sites occupied by vacancies, and *D*_v_ is the vacancy diffusion coefficient. Table 1 shows the parameters used to evaluate Eq. . Figure 5 shows the characteristic grain boundary sintering time as a function of temperature; around the melting point of a 6 nm diameter iron nanoparticle (1483 K) the equivalent grain boundary sintering time is about 2 $\times$ 10^-5^ ns which is significantly less than the estimated viscous sintering time of 0.016 ns for two particles.

**Table 1:** Parameters used to evaluate Eq. (4)

| **Parameter** | **Value** |
| --- | --- |
| *D*_v_ (m^2^/s) | *A*_gb_ exp(−*Q*_v_/(*RT*)) [12]  *A*_gb_ = 5.99^a^  *Q*_v_ = 39750 cal$\cdot$mol^-1 a^  *R* is the gas constant |
| *C*_v0_ (-) | 10^−5^ [13] |
| *γ* (J/m^2^) | 1.826 – (*T*_p_ – *T*_m_) 0.35×10^−3^ [14]  *T*_p_ and *T*_m_ are the particle and melting point temperature, respectively |
| *δ*_gb_ (m) | 10^−6^ [12] |
| *Ω* (m^3^) | (4π/3)*a*^3^  *a* is the atomic radius of Fe taken as 126 pm [15] |

^a^ Taken as the average value of the b.c.c. and f.c.c. lattice structures.

**Figure 5:** Temperature dependence of the characteristic grain boundary sintering time of two iron nanoparticles with equal diameters of 6 nm.

- 1. **Melting point depression**

The melting point depression of the nanoparticles is calculated according to [16]

$T_{m,np}=T_{m,bulk}\left(1-\frac{2}{\rho_{s}\Delta H_{m}r_{p,s}}\left[\gamma_{s}-\gamma_{l}\left(\frac{\rho_{s}}{\rho_{l}}\right)^{2/3}\right]\right)$ ,

where *T*_m,np_ is the melting point of the nanoparticles modified from the bulk value *T*_m,bulk,_ *r*_p,s_ is the radius of the solid particle, *ρ*_s_ and *γ*_s_ are the density and surface tension of the material in the solid phase, and *ρ*_l_ and *γ*_l_ are the density and surface tension of the material in the liquid phase, and Δ*H*_m_ is the enthalpy of fusion. The melting point depression as function of particle diameter is shown in Figure 6.

- 1. **Energy released from sintering**

The energy released during the coalescence of an aggregate is computed by *γ*Δ*A*_s_, where *γ* is the surface energy and Δ*A*_s_ is the change of the surface area from an aggregate morphology to a sphere of equal mass. Using a temperature-dependent surface energy of *γ* [N/m] *=* 1.826 – (*T*_p_ – *T*_m_) 0.35×10^−3^ N$\cdot$m^-1^$\cdot$K^-1^, where *T*_p_ and *T*_m_ are the temperature of the particle and the melting point [14], respectively; and using an aggregate with primary particle diameters ranging from 4–100 nm and a number of primary particles per aggregate of *N*_p_ = 10, 100, 200, 500, and 1000, the released energy is calculated (Figure 6). For an aggregate with *d*_p_ = 6 nm and *N*_p_ = 200, the energy released after coalescence into a sphere is in the order of 10^−14^ J.

**Figure 6:** The magnitude of energy released upon coalescence from aggregates with primary particle diameters ranging from 4–100 nm and N_p_ = 10, 100, 200, 500, and 1000, the energy released after coalescence. The melting point depression as a function of particle diameter is also shown.

1. **LII signal dependence on laser fluence**

Evaluating the heat transfer model for iron nanoparticles with diameters of 10, 15, 20, 25, and 30 nm provides the so-called LII fluence curves shown in Figure 7 that show signs of a plateau at fluences between 10–15 mJ/mm^2^ as opposed to soot nanoparticles that plateau at lower fluences of about 1 mJ/mm^2^ [17,18]. This may be attributed to the lower heat capacity of iron compared to soot which is about 1.8–3.4 times smaller than lower than soot in the temperature range of 300–4000 K.

**Figure 7:** Simulated LII fluence curves for iron nanoparticles with diameters of 10, 15, 20, 25, and 30 nm.

1. **Investigating LII signal contributions from non-thermal emission**

When evaluating the LII signal, potential sources of interference must be considered that can corrupt the evaluation of particle temperatures such as thermally or laser-induced emission of atomic or molecular gas-phase species [19], recombinative chemiluminescence [20], or plasma emission [21,22]. These signals can differ in temporal characteristics depending on excited state lifetimes and formation kinetics of the related excited species. Vander Wal *et al.* [20] experimentally investigated the interference of non-incandescent emissions by analyzing both spectrally- and time-resolved data. In their strategy, they used a spectrograph to determine wavelengths at which only thermal emissions occurred and those that had other emissions and spectral features present. Then, the time-resolved intensities measured at the wavelengths with and without other emissions and spectral features were plotted on a logarithmic scale at different fluences. On the logarithmic scale, they observed that at wavelengths with only thermal emissions, the slopes of the intensities were similar across all fluences and were likely due to evaporative mass loss while the intensities measured at the wavelengths with other emissions present showed a marked change in the slope of the prompt signal as a function of fluence, which they used as an indicator for the presence of a non-LII signal in the time-resolved data. With this strategy, they showed that, for iron nanoparticles irradiated at 1064 nm, increasing amounts of non-thermal emissions interfere with the LII signal at a range of detection wavelengths below 400 nm, although at much higher fluences (> 9 mJ/mm^2^) than those used in our work (≤ 3.14 mJ/mm^2^). It has also been shown for titanium dioxide nanoparticles irradiated at 355 nm that the recorded signals contain LIF, LII, and plasma emissions depending on the laser fluence and also occur at different time scales with LII emissions lasting longer than non-LII emissions [23].

Figure 8a shows that at all fluences investigated in our experiments, the LII signal decay indicates similar particle cooling rates with a rapid decay in the prompt signal that lasts longer at the higher fluences. This is consistent with observations from the literature on irradiated iron [24] and nickel [25] nanoparticles and has been associated with evaporative mass loss. To observe the presence of interfering non-LII signals, the recorded signals are also plotted on a logarithmic scale at selected fluences and wavelengths in Figure 8b, following a procedure by Vander Wal *et al.* [20]. As seen in Figure 8b, for the same detection wavelength, there is no obvious change in the slope of the logarithm of the intensity of the prompt signals as a function of fluence, which, according to the procedure of Vander Wal *et al.* [20], suggests the absence of non-incandescent emissions and the dominance of evaporative cooling. This observation was consistent among all detection wavelengths, and hence, according to Ref. [20], it also follows that no non-incandescent emissions are expected at these wavelengths and evaporative cooling is dominant, however, this hypothesis may be evaluated by streak camera measurements at the fluence ranges used in this experiment.

**Figure 8:** Temporal variation of the LII signal for iron nanoparticles irradiated at varying laser fluence **a.** Linear plot of signal detected at 747 nm. **b.** Semi-logarithmic plot of the measured signal detected at 445 and 747 nm.

1. **Regression the laser heating model to the LII signal**

The current LII model is unable to predict the peak incandescence signal and temperature of iron nanoparticles for a given laser fluence and initial particle morphology even with considering released surface energies. In particular, given the initial temperature of the aerosol and the laser fluence, there are no sets of QoIs including [*d*_p_, *σ*, *α*], that can accurately model the measured signal as shown in Figure 9. This is mostly because there is no size distribution, at a particular fluence, that can model the observed peak temperatures, from which cooling begins. This may be attributed to not properly considering sintering effects in a time-resolved way during laser irradiation which may cause an elevation in peak temperature during the heating of the aerosol.

**Figure 9:** The cooling phase of the regression of the LII model that includes the heating phase, at a fluence of 2.27 mJ/mm^2^, to the data at a *t*_delay_ of **a.** 0 ns, **b.** 100 ns, **c.** 175 ns, and, **d.** 400 ns.

**References**

1. H. A. Michelsen, J. Chem. Phys **118**, 7012 (2003).

2. A. V Filippov, M. W. Markus, and P. Roth, J. Aerosol Sci. **30**, 71 (1999).

3. (n.d.).

4. U. Kreibig and M. Vollmer, *Optical Properties of Metal Clusters* (Springer, 1995).

5. H. Kobatake and H. Fukuyama, Metall. Mater. Trans. A Phys. Metall. Mater. Sci. **47**, 3303 (2016).

6. K. M. Shvarev, B. A. Baum, and P. V. Gel’d, Sov. Phys. J. **18**, 521 (1975).

7. D. W. Mackowski, J. Opt. Soc. Am. A **11**, 2851 (1994).

8. B. T. . Draine and P. J. Flatau, *User Guide for the Discrete Dipole Approximation Code DDSCAT 7.3* (2020).

9. J. Parsons, C. P. Burrows, J. R. Sambles, and W. L. Barnes, J. Mod. Opt. **57**, 356 (2010).

10. M. N. Rahaman, *Sintering of Ceramics* (CRC Press, 2007).

11. M. L. Eggersdorfer, D. Kadau, H. J. Herrmann, and S. E. Pratsinis, J. Aerosol Sci. **46**, 7 (2012).

12. D. W. James and G. M. Leak, Philos. Mag. **12**, 491 (1965).

13. R. W. Siegel, J. Nucl. Mater. **69**–**70**, 117 (1978).

14. B. J. Keene, Int. Mater. Rev. **33**, (1988).

15. J. A. L. Dean, (1999).

16. P. Buffat and J. P. Borel, Phys. Rev. A **13**, 2287 (1976).

17. T. A. Sipkens and K. J. Daun, Opt. Express **25**, 5684 (2017).

18. F. Goulay, P. E. Schrader, X. Lopez-Yglesias, and H. A. Michelsen, Appl. Phys. B Lasers Opt. **112**, 287 (2013).

19. J. Yi, C. Betrancourt, N. Darabiha, and B. Franzelli, Appl. Energy Combust. Sci. **15**, 100190 (2023).

20. R. L. Vander Wal, T. M. Ticich, and J. R. West, Appl. Opt. **38**, 5867 (1999).

21. S. Talebi Moghaddam and K. J. Daun, Appl. Phys. B **124**, 159 (2018).

22. J. Menser, K. Daun, T. Dreier, and C. Schulz, Appl. Opt. **56**, E50 (2017).

23. J. Yi, C. Betrancourt, N. Darabiha, and B. Franzelli, Appl. Phys. B Lasers Opt. **129**, (2023).

24. B. F. Kock, C. Kayan, J. Knipping, H. R. Orthner, and P. Roth, Proc. Combust. Inst. **30**, 1689 (2005).

25. S. Robinson-Enebeli, S. Talebi-Moghaddam, and K. J. Daun, J. Phys. Chem. A **125**, 6273 (2021).
